# Comparator choice in clinical trials of cancer drugs and recommendation categories in National Comprehensive Cancer Network (NCCN) guidelines: a retrospective cohort analysis

**DOI:** 10.1136/bmjopen-2026-122939

**Published:** 2026-10-28

**Authors:** Camille E G Glaus, Cyrill Meier, Simon Hediger, Lara Welti, Kerstin Noelle Vokinger

**Affiliations:** 1 Faculty of Law, University of Zurich, Zurich, Switzerland; 2 Department of Health Sciences and Technology, ETH Zurich, Zurich, Switzerland

**Keywords:** PUBLIC HEALTH, ONCOLOGY, Protocols & guidelines

## Abstract

**Abstract:**

**Background:**

Comparative evidence is important to determine whether new drugs provide added benefit over existing treatments. Ideally, such evidence is generated using appropriate comparators reflecting the standard of care. The National Comprehensive Cancer Network (NCCN) guidelines classify recommendations according to the level of evidence and panel consensus. Category 1 reflects high-level of evidence and categories 2A, 2B and 3 lower-levels of evidence.

**Objective:**

We evaluated how the choice of a comparator was reflected in the NCCN recommendations.

**Methods:**

We conducted a retrospective cohort analysis of cancer drugs approved by the US Food and Drug Administration between 2010 and 2024. For each indication, we identified all populations and assessed whether their corresponding efficacy trials used appropriate, inappropriate or no comparators. We extracted NCCN recommendation categories and compared them across comparator groups. We conducted statistical inference via bootstrap CIs.

**Results:**

Our cohort included 110 indications comprising 176 populations. 56/110 (51%) indications had at least one population tested with an appropriate comparator, 35/110 (32%) had at least one population using an inappropriate comparator, 30/110 (27%) had at least one population based on a single-arm trial and 7/110 (6%) had at least one population for which no data was available. The average proportion of populations classified in NCCN category 1 was 6 percentage points higher with an appropriate than with an inappropriate comparator (95% CI −11 to 25), and 43 percentage points higher than with single-arm trials (95% CI 28 to 58).

**Conclusion:**

Approximately half of the indications recommended in the NCCN guidelines had at least one population tested with an appropriate comparator. The average proportion of NCCN category 1 recommendations did not differ significantly between populations tested with an appropriate and inappropriate comparator but was significantly lower for populations supported by single-arm trials. Greater transparency regarding comparator choice could improve clinical decision-making.

**STRENGTHS AND LIMITATIONS OF THIS STUDY**

- The choice of appropriate comparator was determined based on a systematic method using the Federal Joint Committee reports.
- The Federal Joint Committee is a well-acknowledged German institution whose decisions and documents are also used by other countries and scientists.
- The Federal Joint Committee determines an appropriate comparator in accordance with international standards of evidence-based medicine.
- The cohort was restricted to drugs for which the Federal Joint Committee had determined an appropriate comparator.
- Other appropriate comparators might also be possible.

## Introduction

 For patients and clinicians, it is important to understand whether a new cancer drug is superior, equivalent or inferior to available alternatives. Comparative efficacy evidence generated in clinical trials provides such information.^1^ Ideally, the added clinical benefit of a new drug is tested with the appropriate comparator, that is, the standard of care.^2 3^ Prior studies showed that approximately one-third of new cancer drugs were approved based on single-arm trials and a further proportion relied on trials using an inappropriate comparator with varying estimates going up to almost 50%.^4–9^

The National Comprehensive Cancer Network (NCCN) guidelines, which belong to the most widely used clinical references in oncology for treatment recommendations, take the clinical evidence into account.^10–12^ The recommendation categories depend on the level of evidence and the degree of panel consensus.^10 12 13^ The level of evidence takes into account quality, quantity and consistency of the data from clinical trials.^10 14^ Also other characteristics are considered, such as the appropriateness of a comparator used in the clinical trial of a new cancer drug.^10 14^ NCCN guidelines are annually developed and updated by multidisciplinary expert panels.^10 13 15^

Previous studies analysed how specific aspects of evidence such as accelerated approvals,^16–19^ post-approval trials information,^16 18 20^ reliance on surrogate endpoints were reported in the NCCN guidelines.^18^ Other studies examined the distribution of evidence categories and the underlying data supporting recommendations, including comparisons to therapeutic value framework.^19 21–24^ The question that we address in this study is whether the appropriateness of comparator drugs (appropriate, inappropriate or no comparator) is associated with the NCCN recommendation categories. Ideally, NCCN’s high recommendation category is aligned with cancer drugs that have been tested with appropriate comparators since such comparative efficacy evidence provides patients and clinicians with important information for their decision-making.

## Methods

### Data sources and extraction

We used publicly available databases of the US Food and Drug Administration (FDA) to identify all new cancer drugs (including gene therapies) approved between 1 January 2010 and 31 December 2024.^25^ For each drug, we collected the following information: first indication, approval date and efficacy trials. If the first indication contained multiple indications, all were included in our study cohort. We distinguished between solid and haematologic cancer drugs.

The Federal Joint Committee is a public legal entity under the supervision of the Federal Ministry of Health in Germany that determines the appropriate comparator of a new drug (including one or more targeted populations within a drug), and also publishes the appropriate comparators.^26^ The appropriate comparator corresponds to the standard of care that is routinely used in clinical practice.^27^ It is determined in accordance with international standards of evidence-based medicine.^28^ This information is also used by other countries as well as scientists.^29 30^ We determined for each drug indication whether the efficacy trial used the appropriate comparator based on publicly available information from the Federal Joint Committee.^28^ If the indication consists of multiple populations (eg, mutation or line of treatment), the Federal Joint Committee specifies an appropriate comparator for each population. Examples are outlined in table 1. Based on the assessment reports, we determined whether each population was supported by at least one efficacy trial (pivotal or supportive) that used an appropriate comparator, an inappropriate comparator or with no comparator (ie, single-arm trial). For some populations, no data was reported: These were classified as ‘no data available’. Because differences exist between FDA indications and the populations assessed by the Federal Joint Committee, populations not covered by the corresponding FDA indication were excluded. We excluded drug indications for which the Federal Joint Committee did not determine an appropriate comparator. This included the following cases: the drug indication was not approved in Europe, the sponsor did not submit a dossier to the authority, the evaluation was ongoing or the drug indication had an orphan status in Europe and its annual revenues did not reach €30 million (€50 million up until November 2022).^31–34^ We included the public reports of the Federal Joint Committee through 31 December 2026.

**Table 1:** Examples of drug indications, comparators and NCCN evidence categories

Indication	Population	Appropriate comparator	Evidence	Comparator used	Evaluation	NCCN category
Tislelizumab, as a single agent for the treatment of adult patients with unresectable or metastatic oesophageal squamous cell carcinoma after prior systemic chemotherapy that did not include a PD-(L)1 inhibitor	Same as the indication	Nivolumab	RCT	Physician’s choice of docetaxel, paclitaxel or irinotecan	Inappropriate comparator	1
Encorafenib in combination with binimetinib, for the treatment of patients with unresectable or metastatic melanoma with a BRAF V600E or V600K mutation	Untreated patients	Vemurafenib and cobimetinib or dabrafenib and trametinib	RCT	Vemurafenib	Inappropriate comparator	1
	Previously treated patients	A patient-specific therapy according to the treating physician’s instructions, depending on the respective prior therapy and taking into account the approval status	RCT	Vemurafenib	Inappropriate comparator	2A

NCCN, National Comprehensive Cancer Network; PD-(L)1, programmed cell death protein 1 and its ligand; RCT, randomised controlled trial.

We obtained all NCCN guidelines for the cancer types included in our study cohort. We downloaded the most recent version of each guideline from NCCN’s website and requested the archived versions using the NCCN’s permission request form.^35 36^ We extracted for each drug indication the evidence and consensus of the recommendation at the population level. NCCN recommendations are categorised according to their level of evidence and degree of consensus.^2^ The level of evidence depends on the quality, the quantity and the consistency of the data.^10^ The comparator drug used in the clinical trial may also be considered.^10^ Category 1 reflects high-level of evidence and ≥85% uniform panel consensus, category 2A indicates lower-level evidence and ≥85% uniform panel consensus, whereas category 2B indicates lower-level of evidence and ≥50% until <85% uniform panel consensus. Category 3 includes recommendations based on any level of evidence with major NCCN disagreement.^10^ We extracted the level of evidence and consensus category assigned to each population at the time of its initial inclusion in the guideline.

The populations described in the NCCN recommendations and those in the FDA label did not always align. When an FDA-population was more narrowly defined than the corresponding NCCN recommendation (eg, the FDA label required a specific prior therapy whereas the NCCN recommendation only required a prior line of therapy), it was considered concordant and included. In such cases, multiple populations could correspond to a single NCCN recommendation. When an FDA-population was broader than the NCCN recommendation and could correspond to multiple NCCN recommendations, it was only included if all NCCN recommendations were classified the same category of level of evidence and consensus.

### Statistical analysis

Each population was classified into one of four comparator groups: appropriate comparator, inappropriate comparator, single-arm trial or no data available. Within each drug indication, we calculated the proportion of populations classified in NCCN category 1, 2A, 2B or 3 separately for each comparator group. Then, we averaged these proportions across drug indications within each comparator group. Thus, each drug indication contributed equally, irrespective of the number of populations. A drug indication could contribute to multiple comparator groups if its populations belong to different comparator groups. However, each population was counted only once within its respective comparator group.

We estimated the difference in the average proportion of populations classified in NCCN category 1 between the appropriate and inappropriate comparator groups as well as between the appropriate comparator and the single-arm trial groups. 95% CIs were obtained by a non-parametric cluster bootstrap with drug indications as the resampling unit (1000 resamples; bias-corrected and accelerated intervals, R package boot). In each bootstrap sample, drug indications were drawn with replacement together with all their populations across comparator groups. Group averages and differences were recalculated. This allowed to preserve the correlation of NCCN categories among populations of the same drug indication as well as the dependence between comparator groups arising from drug indications contributing to more than one group. We used the bias-corrected and accelerated bootstrap method because this method has better coverage properties compared with the normal, basic or percentile method.

We conducted a sensitivity analysis distinguishing between solid and haematologic cancer drug indications.

All statistical analyses were conducted using R, V.4.5.1 (R Project for Statistical Computing).

### Patient and public involvement

None.

## Results

### Overview

We identified 173 cancer drugs (190 drug indications) approved by the FDA between 2010 and 2024. Of these, 70 were excluded because the Federal Joint Committee did not determine an appropriate comparator (online supplemental figure S1). The final cohort study comprised 110 drug indications (103 drugs), corresponding to 176 populations. All included populations were recommended in the NCCN guidelines.

### Comparator drug and NCCN recommendations

56/110 (51%) drug indications had at least one population using an appropriate comparator. 35/110 (32%) had at least one population using an inappropriate comparator, and 30/110 (27%) had at least one population approved based on a single-arm trial. 7/110 (6%) included at least one population for which no data was available.

For populations tested with an appropriate comparator, the average proportion classified in NCCN category 1 per drug indication was 46% (56 drug indications), compared with 40% for populations tested with an inappropriate comparator (35 drug indications), 3% for populations based on single-arm trials (30 drug indications) and 14% for populations with no data available (7 drug indications). For populations tested with an appropriate comparator, the average proportion classified in NCCN category 2A per drug indication was 52%, compared with 60% for populations tested with an inappropriate comparator. The average proportion increased to 97% for populations based on single-arm trials and 86% for populations with no data available. No populations in our cohort were classified in NCCN category 2B. One population was classified in NCCN category 3 (figure 1).

**Figure 1:**
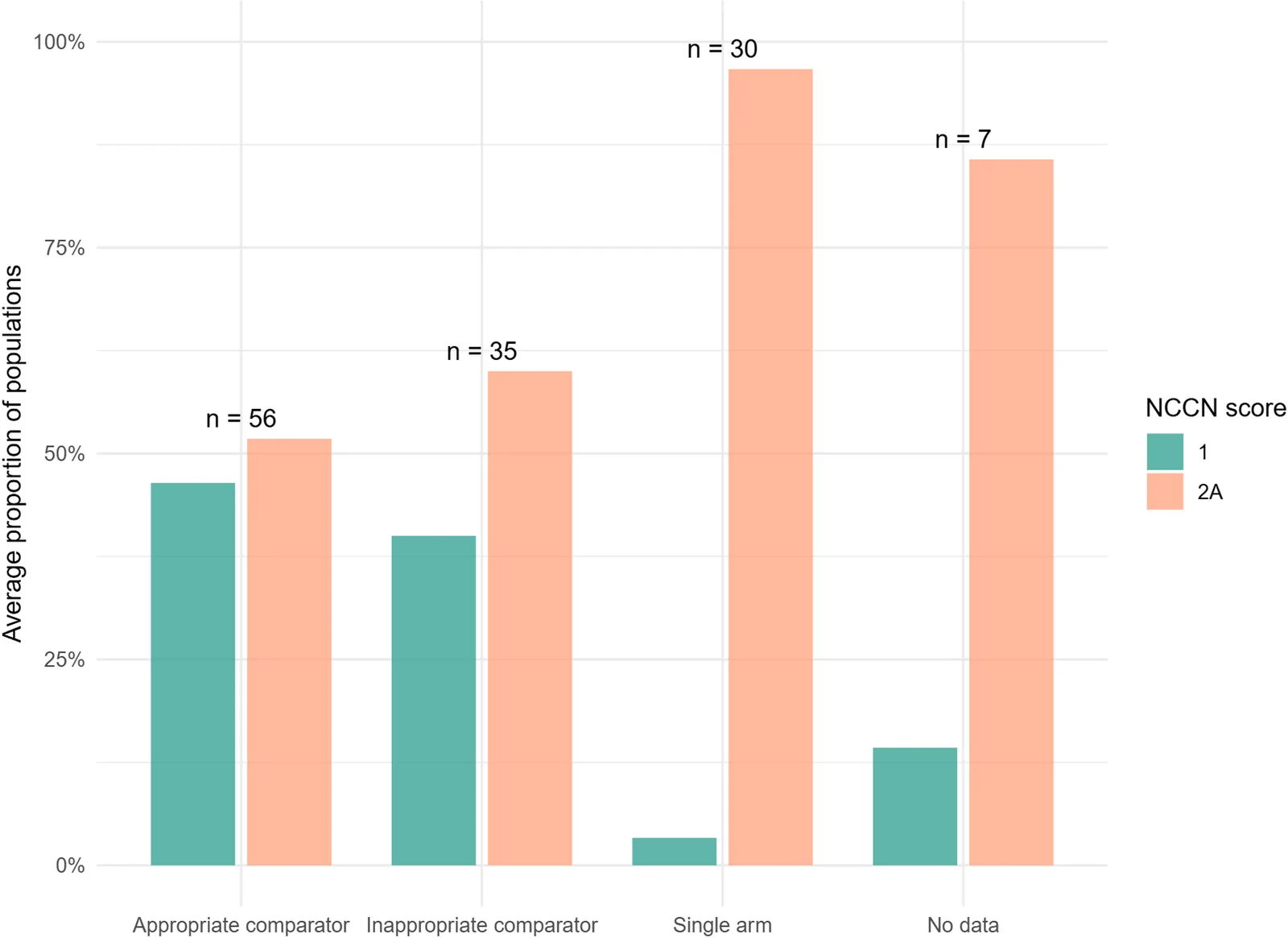
Average percentage of populations within a drug indication classified in NCCN category 1 or 2A across comparator drug groups. Percentages reflect the average proportion of populations within a drug indication classified in each NCCN category for each comparator group and averaged across drug indications. Comparator groups refer to the comparator used for each population and are the following: appropriate comparator, inappropriate comparator, single-arm trials and no data available. A drug indication can contribute to more than one group. NCCN, National Comprehensive Cancer Network.

The average proportion of populations classified in NCCN category 1 was 6 percentage points higher in the appropriate comparator group than in the inappropriate comparator group (95% CI −11 to 25). This difference was not statistically significant. The average proportion was 43 percentage points higher in the appropriate comparator group than in the single-arm trial group (95% CI 28 to 58). The results of the sensitivity analysis were consistent with the primary analysis. For solid tumours, the difference in the average proportion of populations classified in NCCN category 1 between populations tested with an appropriate and an inappropriate comparator was 1 percentage point (95% CI −22 to 18), and between populations tested with an appropriate comparator and populations based on a single-arm trial was 44 percentage points (95% CI 25 to 59). For haematologic malignancies, the corresponding differences were 18 percentage points (95% CI −22 to 53) and 36 percentage points (95% CI 10 to 67), respectively (online supplemental figure S2).

## Discussion

Approximately half of the drug indications recommended in the NCCN guidelines had at least one population tested with an appropriate comparator. We found no statistically significant difference in the average proportion of populations classified in NCCN category 1 between populations tested with an appropriate and an inappropriate comparator. However, we found a significant difference between populations tested with an appropriate comparator and populations based on single-arm trials. The results were similar for the sensitivity analysis.

The choice of comparator is an important evidence information. Clinical trials of cancer drugs tested with appropriate comparators provide an understanding of the additional clinical benefit of a new drug compared with standard of care.^1–3 5 26^ Similarly to our results, previous studies demonstrated that new cancer drugs are not always approved based on randomised controlled trial using an appropriate comparator and thus comparative evidence might be missing.^4–9^ The NCCN panel explicitly acknowledges that comparators may be considered when determining the NCCN category of a recommendation.^11 14^ It appears that recommendations based on single-arm trials were almost automatically categorised in a lower-level of evidence category. However, how and to what extent the comparator is taken into account is not systematically reported. This could explain the absence of a statistically significant difference between populations using an appropriate and inappropriate comparators. This may have consequences in clinical practice as studies showed that physicians tend to overestimate treatment efficacy and underestimate uncertainty of new drugs.^37 38^ Reporting systematically whether the underlying evidence was supported by an appropriate comparator, an inappropriate comparator or a single-arm trial could improve the transparency and the interpretability of NCCN guidelines. This could give physicians a more nuanced understanding of the available evidence and thus guide them better in their clinical decision-making.

Aligned with prior studies, our findings showed that the majority of recommendations were classified in NCCN category 2A, reflecting a lower-level of evidence.^19 21 22 39^ Our results also show heterogeneity within this category among populations approved using different comparators (figure 1). For example, fruquintinib indicated for colorectal cancer was tested against an appropriate comparator, whereas elranatamab approved for multiple myeloma was based on a single-arm trial. Both were classified in the NCCN category 2A. This heterogeneity can be explained by the fact that the choice of comparator is one of several factors considered when evaluating the level of evidence of a recommendation.^10^ Other factors such as the clinical endpoints or the patients of the trial may lower the level of evidence.^10 14^ The influence of comparator choice in NCCN category 2A recommendation is therefore difficult to assess. Adding more granularity within the lower-level of evidence categories could make the NCCN guidelines more informative.^16^

The importance of NCCN category recommendations extends beyond individual clinical decisions.^19^ Concretely, the Center for Medicare and Medicaid Services recognises the NCCN compendium as a reference to make coverage decisions.^40^ Furthermore, the NCCN category recommendations have been incorporated in multiple countries outside the USA (eg, China, Japan, Korea, Russia, Turkey or Latin America) and translated in the respective languages.^12^ Including information in the NCCN guidelines whether an appropriate or inappropriate comparator was included in the clinical trial would in consequence also be helpful for patients and clinicians in countries outside the USA.

### Limitations

Our study has limitations. The Federal Joint Committee did not determine appropriate comparators for all drugs approved in the USA, either because the drug was not approved in Germany or because it had not been assessed by the Federal Joint Committee. Furthermore, it is possible that other scientific or governmental institutions would consider other drugs as appropriate comparators than those defined by the Federal Joint Committee. Also, the populations defined by the Federal Joint Committee did not always correspond exactly to those described in NCCN recommendations. Thus, the findings of our study may not be generalisable to all NCCN recommendations. Finally, these findings relate to NCCN guidelines only and might not be directly generalisable to other guidelines.

## Conclusion

This study found that approximately half of new cancer drug indications recommended by the NCCN included at least a population tested with an appropriate comparator. The proportion of NCCN category 1 recommendations did not differ significantly between populations tested with an appropriate and an inappropriate comparator, whereas they were significantly lower for populations supported by single-arm trials. Incorporating information in the NCCN guidelines whether a new cancer drug was tested based on the appropriate comparator could be informative for patients and clinicians and guide them in their decision-making.

## Supplementary material

10.1136/bmjopen-2026-122939 online supplemental file 1

## Data Availability

Data are available upon reasonable request.
