# Supplementary material for: Comparator choice in clinical trials of cancer drugs and recommendation categories in National Comprehensive Cancer Network (NCCN) guidelines: a retrospective cohort analysis
Source: BMJ Open. 2026 Oct 28;16(10):e122939. doi: 10.1136/bmjopen-2026-122939 (PMC13723834; doi:10.1136/bmjopen-2026-122939)
Supplement: Supplementary file 1 [file bmjopen-16-10-s001.pdf]

Figure S1. Study sample selection process.

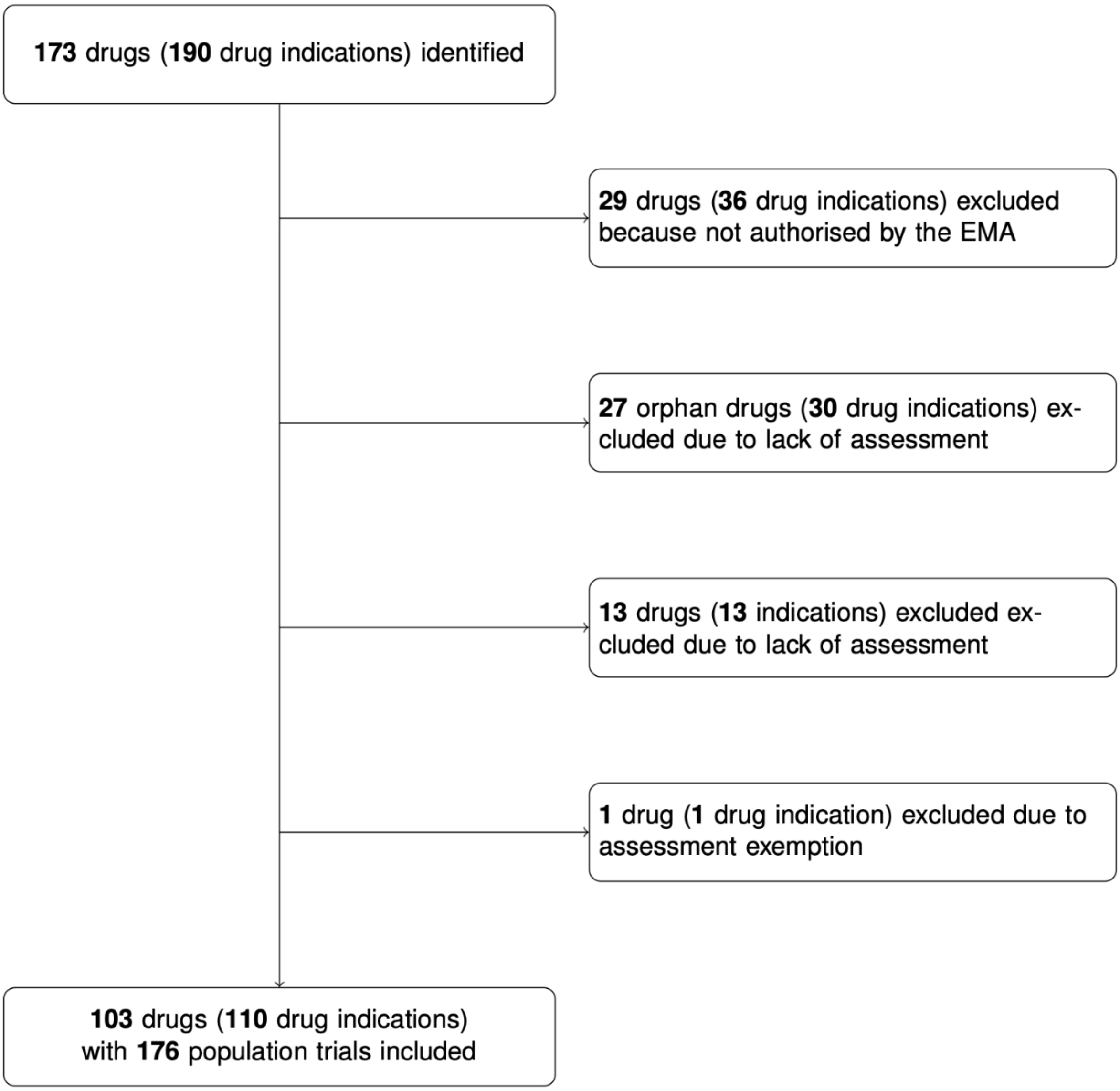

Figure S2. Average percentage of populations within a drug indication classified in NCCN category 1 or 2A across comparator drug groups for haematologic and solid malignancies.

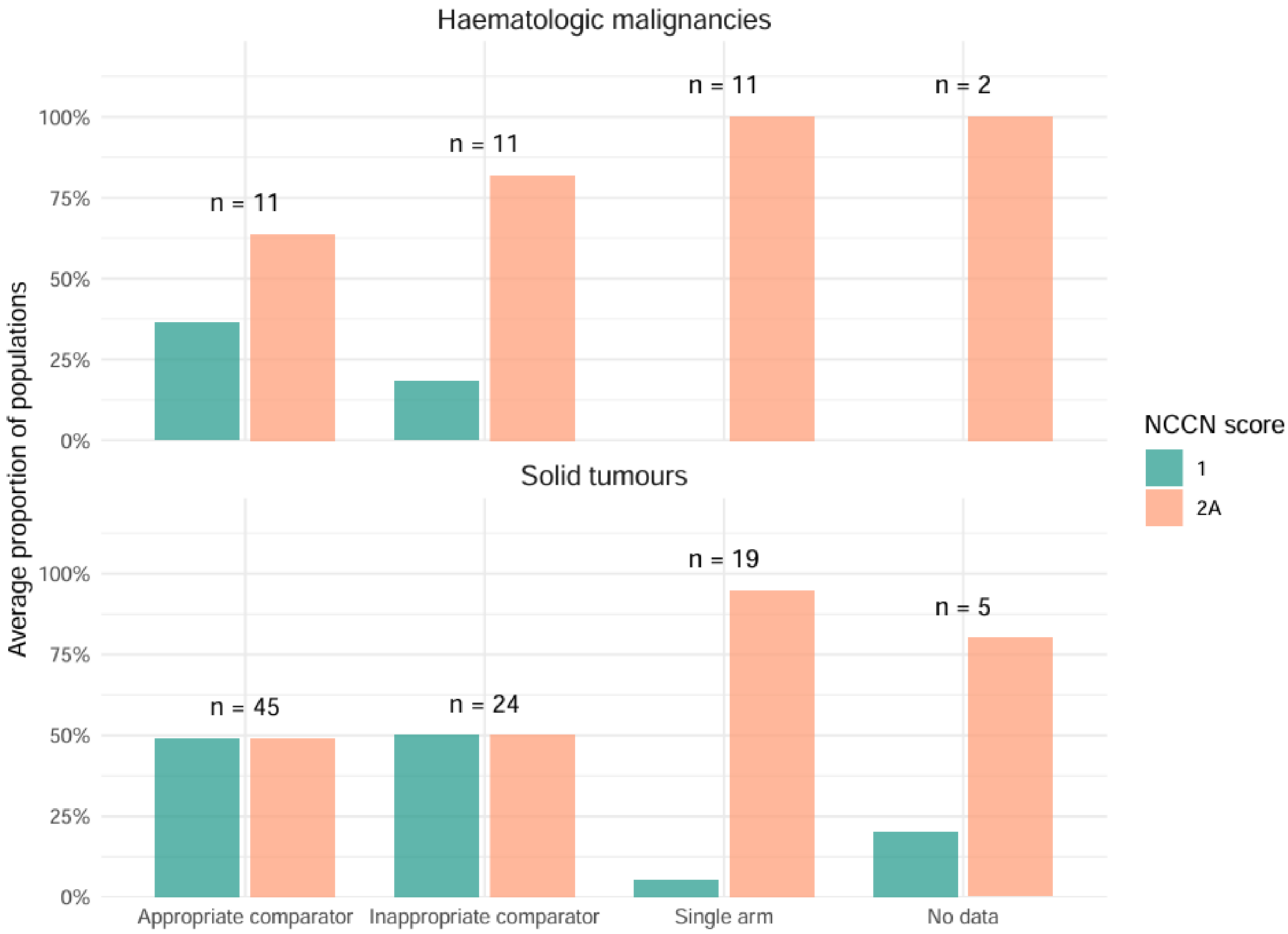

Notes: Percentages reflect the proportion of populations within a drug indication classified in each NCCN category for each comparator group and averaged across drug indications. Comparator groups refer to the comparator used for each population and are the following: appropriate comparator, inappropriate comparator, single-arm trials, and no data available. A drug indication can contribute to more than one group. For solid tumors, the difference in the average proportion of populations classified in NCCN category 1 between populations tested with an appropriate and an inappropriate comparator was 1 percentage point (95% CI −22 to 18), and between populations tested with an appropriate comparator and populations based on a single-arm trial was 44 percentage points (95% CI 25 to 59). For hematologic malignancies, the corresponding differences were 18 percentage points (95% CI −22 to 53) and 36 percentage points (95% CI 10 to 67), respectively.
