# Supplementary material for: Comparator choice in clinical trials of cancer drugs and recommendation categories in National Comprehensive Cancer Network (NCCN) guidelines: a retrospective cohort analysis
Source: BMJ Open. 2026 Oct 28;16(10):e122939. doi: 10.1136/bmjopen-2026-122939 (PMC13723834; doi:10.1136/bmjopen-2026-122939)
Supplement: Supplementary file 2 [file bmjopen-2026-122939.reviewer_comments.pdf]

PEER REVIEW HISTORY

BMJ Open publishes all reviews undertaken for accepted manuscripts. Reviewers are asked to complete a checklist review form and are provided with free text boxes to elaborate on their assessment. These free text comments are reproduced below.

ARTICLE DETAILS

Title (Provisional)

Comparator choice in clinical trials of cancer drugs and recommendation categories in National Comprehensive Cancer Network (NCCN) Guidelines: A retrospective cohort analysis

Authors

Glaus, Camille E.G.; Meier, Cyrill; Hediger, Simon; Welti, Lara; Vokinger, Kerstin Noelle

---

VERSION 1 - REVIEW

**Reviewer**	**1**
**Name**	**Erdat, Efe Cem**
**Affiliation**	**Ankara University, Medical Oncology**
**Date**	**11-Jun-2026**
**COI**	**NA**

This manuscript addresses a relevant question by linking comparator appropriateness (based on German Federal Joint Committee determinations) to NCCN recommendation categories. The topic is worthwhile, but several issues should be addressed.

1. More detailed comparisons are needed. The analysis stays at a fairly high level. The comparisons between groups would benefit from more granular reporting and breakdown rather than the current summary-level presentation.

2. Prevalence figures should appear in the Introduction and Discussion. The estimated percentage of indications relying on inappropriate comparators or single-arm trials should be stated in the Introduction (with reference to prior literature) and revisited in the Discussion, where the cohort's own figures can be compared against existing estimates.

3. Why only NCCN? The rationale for restricting the analysis to the NCCN guidelines should be justified. Considering or at least discussing other guidelines (e.g., ESMO) would strengthen the work and clarify whether the findings are NCCN-specific.

4. NCCN category 2 should be emphasized. The findings around category 2A appear to be where the substance lies — nearly all populations fall into 2A regardless of comparator type. This deserves more emphasis than the category 1 analysis currently receives.

5. Federal Joint Committee exclusions should be detailed. A substantial number of indications were excluded because no appropriate comparator was determined. The reasons and counts for these exclusions should be reported in detail (ideally as a flow diagram), and the potential for selection bias discussed.

6. Table 1 lacks essential information. Table 1 does not include the information that actually demonstrates the classification — notably the comparator that was used in the trial alongside the appropriate comparator, so the reader can see why each indication was judged inappropriate.

7. Figure 1 needs to be modified. The figure is hard to read, and the colour palette significantly affects readability. It should be revised for clarity, with a clearer and more legible colour scheme.

**Reviewer**	**2**
**Name**	**Lu, Yuhan**
**Affiliation department**	**Peking University Cancer Hospital & Institute, Nursing**
**Date**	**23-Jun-2026**
**COI**	**NA**

This study focuses on the selection of comparator in drug clinical trials and high level evidence in NCCN guidelines, exploring the current situation and relationship between the two over the past five years. It is innovative and has positive significance for the quality management of drug clinical trials. However, the following deficiencies still exist

1. Abstract: The presentation of ‘background’ and ‘objective’ is inappropriate, seemingly reversed. Results should include statistical values.

2. The key words of ‘public policy’ is not closely related to this article. It is suggested to replace the keywords which is more in line with the theme.

3. In page 4, “Previous studies analyzed how specific aspects of evidence such as accelerated approvals, post-approval trials information, reliance on surrogate endpoint) were reported in the NCCN guidelines.16–20 ” As the author has summarized the research at different aspects, it is suggested that references be cited separately after the corresponding content instead of listing all of them at the end of the sentence to enhance the readability for readers.

4. Method：“The German Federal Joint Committee is a governmental institution that determines the appropriate comparator of a new drug (including one or more targeted populations within a drug), and also publishes the appropriate comparators.” in page 5. Whether German Federal Joint Committee belongs to a government agency is not very clear. Please consider your expression carefully. Please explain why this institution was chosen as the evaluation criterion and whether there are any other institutions.

5. Statistical method: The author chose bias-corrected and accelerated (BCa) bootstrap confidence intervals. It is suggested to supplement the explanation of the rationality of choosing this method and the applicable conditions.

6. Results：It is recommended to present the statistical results in full, including bias correction factor Z0 and acceleration factor a, to increase the transparency of the methodology in page 10.

7. Discussion: The Discussion content and the Results presentation part should be consistent. The comparison of the single arm is of significant importance, but no relevant discussion content was seen in the Discussion part.

8. The depth of the discussion content is somewhat superficial. It does not conduct an in-depth analysis and discussion of the research results and fails to highlight the practical significance of this research.

9. According to the results of ‘less than half’ and ‘no statistically significant differences between appropriate and inappropriate comparators for the NCCN category 1’, what are the actual phenomena respectively highlighted? What impact does it have on the conduct of drug clinical trials? Please conduct an in-depth analysis and offer insights.

10. Regarding the discussion on heterogeneity in page 11, the recommendation strength of NCCN depends on multiple aspects, and the source of heterogeneity is not entirely the selection of comparators. Does the comparators have any impact on other aspects? It is suggested that the author discuss the sources of heterogeneity.

11. References：References [26] and [30] are repeated, please proofread. References [31-34] are all in German. It is necessary to supplement their English translations to facilitate readers' understanding.

VERSION 1 - AUTHOR RESPONSE

Reviewer 1	Author response and changes made	Page number in revised paper

<table>
<tr>
<td>This manuscript addresses a relevant question by linking comparator appropriateness (based on German Federal Joint Committee determinations) to NCCN recommendation categories. The topic is worthwhile, but several issues should be addressed.</td>
<td>Thank you very much for your comments and your insightful feedback.</td>
<td></td>
</tr>
<tr>
<td>1. More detailed comparisons are needed. The analysis stays at a fairly high level. The comparisons between groups would benefit from more granular reporting and breakdown rather than the current summary-level presentation.</td>
<td>Thank you for this valuable comment. We conducted an additional analysis to determine whether our results hold when distinguishing between different groups. Given the limited number of observations of our cohort, we classified our data in two groups: hematologic and solid cancer drugs.

We observed similar results in both groups, consistent with our main analysis. Specifically, we observed no significant differences in the average percentage of populations assigned to NCCN category 1 between drug indications with at least one population tested with an appropriate comparator and those with at least one population tested with an inappropriate comparator. In contrast, there were statistically significant differences in the average percentage of populations assigned to NCCN category 1 between drug indications with at least one population tested with an appropriate comparator and those with at least one population approved based on a single-arm trial. We provide you with a small table that summarizes the results of our additional analysis.

<table>
<tr><th></th><th>Comparison</th><th>Difference</th><th>95%CI</th></tr>
<tr><td>Solid tumor</td><td>appropriate vs. inappropriate</td><td>1%</td><td>-22% to 18%</td></tr>
<tr><td>Solid tumor</td><td>appropriate vs. single-arm trial</td><td>44%</td><td>25% to 59%</td></tr>
<tr><td>Hematologic malignancies</td><td>appropriate vs. inappropriate</td><td>18%</td><td>-22% to 53%</td></tr>
<tr><td>Hematologic malignancies</td><td>appropriate vs. single-arm trial</td><td>36%</td><td>10% to 67%</td></tr>
</table>

We created a figure (Figure S2), in the same style as Figure 1, to illustrate those findings. Figure S2 is part of the supplemental materials.

Besides, we modified our method section, our result section and the discussion.

Specifically, in the methods section we added the following sentences:

“We distinguished between solid and hematological cancer drugs.” And “We conducted a sensitivity analysis</td>
<td>5, 8</td>
</tr>
</table>

	distinguishing between solid and hematologic cancer drugs”.<br><br>In the result section, we added the following paragraph:<br><br>The results of the sensitivity analysis were consistent with the primary analysis. For solid tumors, the difference in the average percentage of populations assigned to NCCN category 1 between drug indications with at least one population tested with an appropriate comparator compared to those with at least one population tested with an inappropriate comparator was 1% (95% CI: −22% to 18%), while the difference between drug indications with at least one population tested with an appropriate comparator compared to those with at least one population based on a single-arm trial was 44% (95% CI: 25% to 59%). For hematologic malignancies, the corresponding differences were 18% (95% CI: −22% to 53%) and 36% (95% CI: 10% to 67%), respectively (Figure S2).<br><br>In the *discussion* section, we added the following information:<br><br>“We found similar results for the sensitivity analysis” and<br><br>“This is also the case when distinguishing between hematologic and solid cancer drug indications.”	
2. Prevalence figures should appear in the Introduction and Discussion. The estimated percentage of indications relying on inappropriate comparators or single-arm trials should be stated in the Introduction (with reference to prior literature) and revisited in the Discussion, where the cohort's own figures can be compared against existing estimates.	Thank you for this valuable point. We contextualized our paper within the existing literature. The estimates of other studies were slightly different. Other studies found that around a third of cancer drugs are approved based on single-arm trials (4). The proportions of drugs based on a trial using an appropriate comparator vary across studies. Several factors may account for these differences.<br><br>First, the methodology differed depending on the publication. Some studies conducted their analysis at the indication level only and ignored the different patient populations (for instance: (6) or (8)). Besides, we determined the appropriate comparator based on a consistent and systematic methodology (using the Federal Joint Committee documents) while other studies manually searched publications and meta-analyses (for example (6)). Further, the study periods are not identical. This has consequences: not only are the drug cohorts different but also the regulatory standards and the evidentiary requirements may change overtime. Finally, our cohort was limited to FDA approved cancer (first indications) for which the German Federal Joint Committee has conducted an evaluation. Other studies included supplemental indication, analyzed accelerated approval drugs only or focused on specific cancer types (for example (7)).<br><br>For all these reasons, it is quite delicate to conduct a direct comparison that is fair. Nevertheless, we revised our manuscript to incorporate some results of these studies.	4, 11

	Concretely, we modified the introduction and the discussion. To avoid overburdening the reader, we decided to keep the comparison between our results and the literature relatively concise.<br><br>In the *introduction* we modified the following sentence: "Prior studies showed that many new cancer drugs were approved based on single-arm trials" to "Prior studies showed that approximately one-third of new cancer drugs were approved based on single-arm trials and a further proportion relied on trials using an inappropriate comparator with varying estimates going up to almost 50%.[4-9]"<br><br>In the *discussion* we added the following sentence: "Similarly to our results, previous studies demonstrated that new cancer drugs are not always approved based on randomized controlled trial using an appropriate comparator and thus comparative evidence is missing.[4-9]"<br><br>(4) Agrawal S, Arora S, Amiri-Kordestani L, et al. Use of Single-Arm Trials for US Food and Drug Administration Drug Approval in Oncology, 2002-2021. JAMA Oncol. 2023;9(2):266. doi:10.1001/jamaoncol.2022.5985<br>(5) Glaus CEG, Herrle C, Hediger S, Vokinger KN. Comparative efficacy evidence of new drugs (2011-2024) – Retrospective cohort study on the use of appropriate, inappropriate and no comparators in efficacy trials.<br>(6) Hilal T, Sonbol MB, Prasad V. Analysis of Control Arm Quality in Randomized Clinical Trials Leading to Anticancer Drug Approval by the US Food and Drug Administration. JAMA Oncol. 2019;5(6):887. doi:10.1001/jamaoncol.2019.0167<br>(7) Hilal T, Mohyudin A, Ayala R, Almader-Douglas D, Leis JF. Quality of the control arms in randomized trials of chronic lymphocytic leukemia enrolling in the USA: A systematic review. European Journal of Cancer. 2023;180:4-8. doi:10.1016/j.ejca.2022.11.024<br>(8) Mohyuddin GR, Koehn K, Sborov D, et al. Quality of control groups in randomised trials of multiple myeloma enrolling in the USA: a systematic review. The Lancet Haematology. 2021;8(4):e299-e304. doi:10.1016/S2352-3026(21)00024-7<br>(9) Rossi A, Aimar G, Audisio M, et al. Analysis of the adequacy of control arms in oncology randomised clinical trials published between 2017 and 2021: a meta-research study. European Journal of Cancer. 2023;189:112920. doi:10.1016/j.ejca.2023.05.008	
3. Why only NCCN? The rationale for restricting the analysis to the NCCN guidelines should be justified. Considering or at least discussing other guidelines (e.g., ESMO) would strengthen	We thank the reviewer for this important point. We agree that several other guidelines such as the mentioned ESMO guidelines play an important role in clinical practice.<br><br>We chose to focus on the NCCN guidelines for several reasons. First, the NCCN guidelines cover a wide range of	12

the work and clarify whether the findings are NCCN-specific.	cancer types and offer indication-specific recommendations. Second, they are one of the most widely used guidelines in oncology. Third, they are used as a reference to make coverage decisions by the Center for Medicare and Medicaid Services in the US. Fourth, they are also used internationally with specific international adaptations.<br><br>In this study, our objective was not to compare recommendations across different guidelines but rather to focus specifically on the NCCN guidelines. Restricting to the NCCN guidelines allowed an in-depth analysis based on a consistent methodology. We agree with the reviewer that our findings might not be generalizable as there are differences across guidelines.<br><br>We highlighted this point in the *limitation* section of our manuscript with the following sentence:<br>"Finally, these findings relate to NCCN guidelines only and might not be directly generalizable to other guidelines."	
4. NCCN category 2 should be emphasized. The findings around category 2A appear to be where the substance lies — nearly all populations fall into 2A regardless of comparator type. This deserves more emphasis than the category 1 analysis currently receives.	Thank you for pointing this out. As we mentioned in the discussion, our findings align with other studies that specifically analyzed this point (for example: (1)).<br><br>We believe that drawing specific conclusions between these findings (most recommendations falling under NCCN category 2) and the use of an appropriate comparator is delicate because category 2A includes all drugs based on a "lower-level of evidence". The choice of comparator is only one aspect of level of evidence. Multiple factors beyond the comparator choice may lead to a recommendation being classified as NCCN category 2A, 2B or 3. A recommendation may be classified in NCCN category 2A because of the clinical endpoint or the patient population of the clinical trial. Actually, the NCCN guidelines state that all recommendations default to NCCN category 2A unless otherwise noted. In contrast, NCCN category 1 requires a higher-level of evidence. The choice of an appropriate comparator is an important element to evaluate the level of evidence and one could argue that only recommendations based on an appropriate comparator should be classified in NCCN category 1.<br><br>Based on this comment, we revised the *discussion.* Concretely, we added the following sentences:<br>"This heterogeneity can be explained by the fact that the choice of comparator is one of several factors considered when evaluating the level of evidence of a recommendation.[10] Other factors such as the clinical endpoints or the patients of the trial may lower the level of evidence.[10,14] The influence of comparator choice in NCCN category 2A recommendation is therefore difficult to assess. Adding more granularity within the lower-level of evidence categories could make the NCCN guidelines more informative.[16]"	9, 11-12

	(1) Kurzrock R, Gurski LA, Carlson RW, et al. Level of evidence used in recommendations by the National Comprehensive Cancer Network (NCCN) guidelines beyond Food and Drug Administration approvals. Annals of Oncology. 2019<br><br>(10) National Comprehensive Cancer Network. Development and Update of Guidelines. https://www.nccn.org/guidelines/guidelines-process/development-and-update-of-guidelines<br><br>(14) McClure JS. Evidence and Insights in the NCCN Guidelines. J Natl Compr Canc Netw. 2012;10(4):427-429. doi:10.6004/jnccn.2012.0043<br><br>(16) Cliff ERS, Rome RS, Kesselheim AS, Rome BN. National Comprehensive Cancer Network Guideline Recommendations of Cancer Drugs With Accelerated Approval. JAMA Netw Open. 2023;6(11):e2343285. doi:10.1001/jamanetworkopen.2023.43285	
5. Federal Joint Committee exclusions should be detailed. A substantial number of indications were excluded because no appropriate comparator was determined. The reasons and counts for these exclusions should be reported in detail (ideally as a flow diagram), and the potential for selection bias discussed.	We agree with the reviewer. As we mentioned in our method section, there are multiple reasons leading to exclusions of drugs. First, some of the drugs are not approved by the EMA (refused, application withdrawn, application never submitted or time lag between FDA and EMA). Second, the drug has to be assessed by the German Federal Joint Committee. There a several situations in which the drug will not be assessed: the drug is not marketed in Germany, the sponsor does not submit a dossier to the Federal Joint Committee or the drug is exempt from evaluation (e.g., orphan drugs with annual revenues below EUR 30 million) or the Federal Joint Committee has not evaluated the drug yet. We tried to mitigate this last problem by considering all the Federal Joint Committee assessments until the end of 2025. We added a sentence in our *methods* section to clarify this point: “We included the public reports of the German Federal Joint Committee through 31 December 2025.”<br><br>Additionally, we created an additional flow diagram as a supplemental figure (Figure S1).<br><br>We do not expect a systematic bias due to the following reasons: First, our policy implications aims to apply to drugs that were approved by the EMA and assessed by the Federal Joint Committee and not to those approved by the EMA but not assessed by the Federal Joint Committee. Second, the choice of comparator is not a condition per se for the approval of a drug by the EMA (1). At least based on approval requirements, there is no systematic reason for a drug to be approved by the FDA and not by the EMA. Therefore, we do not think that our conclusions are impacted by a selection bias.<br><br>However, we clarified our *limitation* section. We changed the sentence:<br><br>“The Federal Joint Committee did not determine	Figure S1, 12-13

	appropriate comparators for all drugs approved in the US" to "The Federal Joint Committee did not determine appropriate comparators for all drugs approved in the US either because the drug was not approved in Germany or because it had not been assessed by the Federal Joint Committee".<br><br>Besides, we also listed this limitation as one of the limitations of our study in the category "*strengths and limitations of this study*". We wrote:<br>"The cohort was restricted to drugs for which the Federal Joint Committee had determined an appropriate comparator."<br><br>(1) Naci H, Salcher-Konrad M, Kesselheim AS, Wieseler B, Rochaix L, Redberg RF, Salanti G, Jackson E, Garner S, Stroup TS, Cipriani A. Generating comparative evidence on new drugs and devices before approval. Lancet. 2020 Mar 21;395(10228):986-997. doi: 10.1016/S0140-6736(19)33178-2.	
6. Table 1 lacks essential information. Table 1 does not include the information that actually demonstrates the classification — notably the comparator that was used in the trial alongside the appropriate comparator, so the reader can see why each indication was judged inappropriate.	This is a good point raised by the reviewer. We added two additional columns, one indicating which comparator was used in the clinical trial (column "Comparator used") and the other indicating whether the comparator used was appropriate or inappropriate (column "Evaluation").	Table 1 (p. 19).
7. Figure 1 needs to be modified. The figure is hard to read, and the colour palette significantly affects readability. It should be revised for clarity, with a clearer and more legible colour scheme.	We changed the color palette to make the figure easier to read.	Figure 1

Reviewer 2

Reviewer 2	Author response and changes made	Page number in revised paper
This study focuses on the selection of comparator in drug clinical trials and highlevel evidence in NCCN guidelines, exploring the current situation and relationship between the two over the past five years. It	Thank you very much.	

is innovative and has positive significance for the quality management of drug clinical trials. However, the following deficiencies still exist.		
1.Abstract: The presentation of 'background' and 'objective' is inappropriate, seemingly reversed. Results should include statistical values.	We revised our abstract and included some statistical values. More particularly we revised in depth the background and objective parts of the *abstract*. It now read as follow:<br><br>"**Background:** Comparative evidence is important to determine whether new drugs provide added benefit over existing treatments. Ideally, such evidence is generated using appropriate comparators reflecting the standard of care. The National Comprehensive Cancer Network (NCCN) guidelines classify recommendations according to the level of evidence and panel consensus. Category 1 reflects high-level of evidence and categories 2A, 2B and 3 lower-levels of evidence.<br><br>**Objective**: We evaluated how the choice of an appropriate comparator was reflected in the NCCN recommendations."<br><br>We also added the statistical values in the sentence: "There was no statistically significant difference (95% CI: -11% to 25%) in the average percentage of patient populations assigned to NCCN category 1 between indications that had at least one patient population using an appropriate comparator and those using at least one inappropriate comparator»<br><br>We also changed slightly a sentence in the *methods* section of the abstract that we believe better explain what we did. The prior sentence was:<br>"We extracted NCCN recommendation categories (1, 2A, 2B or 3). For each patient population, we analyzed the association between comparator types and NCCN category recommendation. Differences were assessed using bootstrap confidence intervals". We changed it to "We extracted NCCN recommendation categories and compared them across comparator groups. We conducted statistical inference via bootstrap confidence intervals."<br><br>Finally, we changed a few words overall to make sure we stay within the maximum number of words allowed (300 words).	2
2.The key words of 'public policy' is not closely related to this article. It is suggested to replace the keywords which is more in line with the theme.	We replaced the keyword 'public policy' with 'NCCN recommendations'.	2
3.In page 4, "Previous studies analyzed how specific aspects of evidence such as accelerated approvals, post-approval trials information, reliance on surrogate	We modified the references accordingly, that is:<br><br>"Previous studies analyzed how specific aspects of evidence such as accelerated approvals,[16–19] post-approval trials information,[16,18,20] reliance on surrogate endpoint) were	4

endpoint) were reported in the NCCN guidelines.16–20 " As the author has summarized the research at different aspects, it is suggested that references be cited separately after the corresponding content instead of listing all of them at the end of the sentence to enhance the readability for readers.	reported in the NCCN guidelines.[18]"	
4.Method "The German Federal Joint Committee is a governmental institution that determines the appropriate comparator of a new drug (including one or more targeted populations within a drug), and also publishes the appropriate comparators." in page 5. Whether German Federal Joint Committee belongs to a government agency is not very clear. Please consider your expression carefully. Please explain why this institution was chosen as the evaluation criterion and whether there are any other institutions.	The German Federal Joint Committee is a public legal entity that is under the supervision of the Federal Ministry of Health (https://www.g-ba.de/english/structure/?utm_source=chatgpt.com). We modified our manuscript as such: instead of "The German Federal Joint Committee is a governmental institution that determines the appropriate comparator of a new drug (including one or more targeted populations within a drug), and also publishes the appropriate comparators", we wrote "The German Federal Joint Committee is a public legal entity under the supervision of the Federal Ministry of Health that determines the appropriate comparator of a new drug (including one or more targeted populations within a drug), and also publishes the appropriate comparators"<br><br>We chose the Federal Joint Committee assessments for multiple reasons.<br><br>First, few institutions determine the appropriate comparator of new drugs and share it publicly. Other institutions such as the Haute Autorité de Santé in France or NICE in England discuss the appropriate comparator but the Federal Joint Committee does it very systematically. Then, the Federal Committee is a well acknowledged institution. Their decisions and documents are used by other countries and scientists. Their methodology (including the choice of an appropriate comparator) has already been used in the new European Health Technology Assessment (https://health.ec.europa.eu/health-technology-assessment/key-documents_en?f%5B0%5D=topic_topic%3A247). Finally, the Federal Joint Committee chooses the appropriate comparator based on international standards of evidence-based medicines. Thus, it very likely reflects what the standard of care is. We added the following sentence in our manuscript to reflect this particular point: "It is determined in accordance with international standards of evidence-based medicine.[28]"<br><br>We modified the section '*strengths and limitations of this study'* to reflect these points. We added the following two bullet points:<br><br>"The Federal Joint Committee is a well-acknowledged institution whose decisions and documents are used by other countries and scientists" and	3, 5

	"The Federal Joint Committee determines an appropriate comparator in accordance with international standards of evidence-based medicine".<br><br>(28) Gemeinsamer Bundesausschuss (G-BA). Rules of Procedure of the Federal Joint Committee. 2026. https://www.g-ba.de/downloads/62-492-4105/VerfO_2025-11-20_iK_2026-04-08.pdf	
5.Statistical method: The author chose bias-corrected and accelerated (BCa) bootstrap confidence intervals. It is suggested to supplement the explanation of the rationality of choosing this method and the applicable conditions.	Thank you for this point. Dr. Hediger ran an additional basic bootstrap. The results stayed very similar.<br><br>We decided to keep the bias-corrected and accelerated bootstrap method because this method is known to have better coverage properties compared to the normal, basic or percentile method.	-
6.Results : It is recommended to present the statistical results in full, including bias correction factor Z0 and acceleration factor a, to increase the transparency of the methodology in page 10.	The bias correction factor is -0.0289 and the acceleration factor is -0.0036. Both numbers are very close to zero indicating no severe bias or skewness.<br><br>If the reviewer also agrees, we would prefer not to report these figures because they are algorithmic internal parameters (not estimated quantities of interest).	-
7.Discussion: The Discussion content and the Results presentation part should be consistent. The comparison of the single arm is of significant importance, but no relevant discussion content was seen in the Discussion part.	We agree with the reviewer that our discussion focussed mainly on appropriate vs inappropriate comparator and that our results regarding single-arm trials were not clearly stated.<br><br>We modified our *discussion* section to better reflect these results. Concretely, we added the following sentence:<br>"In contrast, our results indicate that recommendations supported by single-arm trials were less likely to be classified in NCCN category 1."<br><br>We revised the sentence:<br>"The NCCN panel explicitly acknowledges that comparators may be considered when determining the NCCN category of a recommendation.[11,14] How and to what extent the comparator should be taken into account is not systematically reported. This could be one possible explanation why the level of evidence is not aligned with the appropriateness of the comparator drug." to<br>"The NCCN panel explicitly acknowledges that comparators may be considered when determining the NCCN category of a recommendation.[11,14] It appears that recommendations based on single-arm trials were almost automatically categorized in a lower-level of evidence category. However, how and to what extent the comparator is taken into account is not systematically reported. This could be one possible explanation why the level of evidence is not aligned with the appropriateness of the comparator drug."	11-13

<table>
<tr>
<td></td>
<td>
We also revised the sentence:
“Reporting systematically whether an appropriate or inappropriate comparator was included in the clinical trial could improve the transparency and the interpretability of NCCN guidelines» to
“Reporting systematically whether the underlying evidence was supported by an appropriate comparator, an inappropriate comparator or a single-arm trial could improve the transparency and the interpretability of NCCN guideline. This could give physicians a more nuanced understanding of the available evidence and thus better guide them in their clinical decision-making.”
(11) National Comprehensive Cancer Network. About Clinical Practice Guidelines. https://www.nccn.org/guidelines/guidelines-process/about-nccn-clinical-practice-guidelines. (Accessed 21 July 2026)
(14) McClure JS. Evidence and Insights in the NCCN Guidelines. J Natl Compr Canc Netw. 2012;10(4):427-429. doi:10.6004/jnccn.2012.0043
</td>
<td></td>
</tr>
<tr>
<td>8.The depth of the discussion content is somewhat superficial. It does not conduct an in-depth analysis and discussion of the research results and fails to highlight the practical significance of this research.</td>
<td>
We revised our manuscript to give it more depth.
First, we conducted a sensitivity analysis for hematologic malignancies and solid cancer drug indications.
We observed similar results in both groups, consistent with our main analysis. Specifically, we observed no significant differences in the average percentage of populations assigned to NCCN category 1 between drug indications with at least one population tested with an appropriate comparator and those with at least one population tested with an inappropriate comparator. In contrast, there were statistically significant differences in the average percentage of populations assigned to NCCN category 1 between drug indications with at least one population tested with an appropriate comparator and those with at least one population approved based on a single-arm trial. We provide you with a small table that summarizes the results of our additional analysis.
<table>
<tr><th></th><th>Comparison</th><th>Difference</th><th>95%CI</th></tr>
<tr><td>Solid tumor</td><td>appropriate vs. inappropriate</td><td>1%</td><td>-22% to 18%</td></tr>
<tr><td>Solid tumor</td><td>appropriate vs. single-arm trial</td><td>44%</td><td>25% to 59%</td></tr>
<tr><td>Hematologic malignancies</td><td>appropriate vs. inappropriate</td><td>18%</td><td>-22% to 53%</td></tr>
<tr><td>Hematologic malignancies</td><td>appropriate vs. single-arm trial</td><td>36%</td><td>10% to 67%</td></tr>
</table>
We created a figure (Figure S2), in the same style as Figure 1, to illustrate those findings. Figure S2 is part of the
</td>
<td>11-13</td>
</tr>
</table>

	supplemental materials.<br><br>Besides, we modified our method section, our result section and the discussion.<br><br>Specifically, in the *methods* section we added the following sentences:<br><br>“We distinguished between solid and hematological cancer drugs.” And “We conducted a sensitivity analysis distinguishing between solid and hematologic cancer drugs”.<br><br>In the result section, we added the following paragraph:<br><br>The results of the sensitivity analysis were consistent with the primary analysis. For solid tumors, the difference in the average percentage of populations assigned to NCCN category 1 between drug indications with at least one population tested with an appropriate comparator compared to those with at least one population tested with an inappropriate comparator was 1% (95% CI: −22% to 18%), while the difference between drug indications with at least one population tested with an appropriate comparator compared to those with at least one population based on a single-arm trial was 44% (95% CI: 25% to 59%). For hematologic malignancies, the corresponding differences were 18% (95% CI: −22% to 53%) and 36% (95% CI: 10% to 67%), respectively (Figure S2).<br><br>In the *discussion* section, we added the following information:<br><br>“We found similar results for the sensitivity analysis” and<br><br>“This is also the case when distinguishing between hematologic and solid cancer drug indications.”<br><br>Second, we developed the paragraph recommendations of NCCN category 2A (third paragraph of the discussion) in the *discussion*. We believe that NCCN category 1 should reflect the use of an appropriate comparator because the use of an appropriate comparator is an important information of evidence and NCCN category 1 recommendations are high-level of information (second paragraph of the discussion). However, a link between the use of an appropriate comparator and a NCCN category 2 is more difficult to make. In our manuscript, we gave the example of fruquitinib that was approved based on appropriate comparator but was still categorized in NCCN category 2A. For this specific case it is difficult to say why, whether and how the choice of comparator was relevant. Other factors probably influenced the recommendation category. We reflected this argument by adding these sentences in our *discussion*:<br><br>“This heterogeneity can be explained by the fact that the choice of comparator is one of several factors considered when evaluating the level of evidence of a recommendation.[10] Other factors such as trial endpoints or patient populations may lower the level of evidence.[10,14] The influence of comparator choice in NCCN category 2A recommendation is therefore difficult to assess. Adding more granularity within the lower-level of evidence categories could make the NCCN guidelines more informative.[16]”	

	Third, we clarified and better highlighted the practical significance of our research. In the *discussion* section we modified the sentences<br><br>“studies showed that physicians tend to overestimate treatment efficacy and underestimate uncertainty of new drugs.[37,38] Reporting systematically whether an appropriate or inappropriate comparator could improve the transparency and the interpretability of NCCN guidelines” to<br><br>“This may have consequences in practice as studies showed that physicians tend to overestimate treatment efficacy and underestimate uncertainty of new drugs.[37,38] Reporting systematically whether the underlying evidence was supported by an appropriate comparator, an inappropriate comparator or a single-arm trial could improve the transparency and the interpretability of NCCN guideline. This could give physicians a more nuanced understanding of the available evidence and thus better guide them in their clinical decision-making.”	
9.According to the results of ‘less than half’ and ‘no statistically significant differences between appropriate and inappropriate comparators for the NCCN category 1’, what are the actual phenomena respectively highlighted? What impact does it have on the conduct of drug clinical trials? Please conduct an in-depth analysis and offer insights.	Our main message is twofold.<br><br>First, comparative evidence with an appropriate comparator is often missing (‘less than half’). Concretely, it means that cancer drugs are approved without a clear understanding of how well they performed compared to the standard of care. Multiple studies have documented this factor in different contexts (for example: 1, 2, 3). Second, NCCN category 1 does not seem to systematically consider the choice of an appropriate comparator. Concretely, it means that recommendations are based on limited evidence and this is not necessarily reflected in NCCN category 1.<br><br>Regarding the impact on clinical trial conduct, our findings only indicate that clinical trials are not necessarily designed to use an appropriate comparator. Several factors may explain this such as costs and time constraints, difficulty of patient recruitment, lack of consensus on what constitutes an appropriate comparator or lack of incentives to use an appropriate comparator. A comprehensive analysis of these factors goes beyond the scope of our study and would prefer not to develop it further in this piece. However, we have another publication that is currently under review that analyses the use of appropriate comparators for new drugs that explores the consequences of these choices.<br><br>(1) Van Luijn JCF, Gribnau FWJ, Leufkens HGM. Superior efficacy of new medicines? *Eur J Clin Pharmacol*. 2010;66(5):445-448. doi:10.1007/s00228-010-0808-3<br><br>(2) Agrawal S, Arora S, Amiri-Kordestani L, et al. Use of Single-Arm Trials for US Food and Drug Administration Drug Approval in Oncology, 2002-2021. *JAMA Oncol*. 2023;9(2):266. doi:10.1001/jamaoncol.2022.5985<br><br>(3) Hilal T, Sonbol MB, Prasad V. Analysis of Control Arm Quality in Randomized Clinical Trials Leading to Anticancer Drug Approval by the US Food and Drug Administration.	-

	JAMA Oncol. 2019;5(6):887. doi:10.1001/jamaoncol.2019.0167	
10.Regarding the discussion on heterogeneity in page 11, the recommendation strength of NCCN depends on multiple aspects, and the source of heterogeneity is not entirely the selection of comparators. Does the comparators have any impact on other aspects? It is suggested that the author discuss the sources of heterogeneity.	As mentioned under the point 8, we agree with the reviewer that the recommendation strength of the NCCN recommendations depends on different aspects beyond the choice of an appropriate comparator. As mentioned above, we modified the *discussion* section. We made nuances clearer for the reader we added the following:<br><br>"This heterogeneity can be explained by the fact that the choice of comparator is one of several factors considered when evaluating the level of evidence of a recommendation.[10] Other factors such as trial endpoints or patient populations may lower the level of evidence.[10,14] The influence of comparator choice in NCCN category 2A recommendation is therefore difficult to assess. Distinguishing more precisely among the lower-level of evidence could make the NCCN guidelines more informative.[16]"	11-12
11.References : References [26] and [30] are repeated, please proofread. References [31-34] are all in German. It is necessary to supplement their English translations to facilitate readers' understanding.	Thank you for spotting this. We modified the references accordingly and translated the titles in English. However, some of the documents we cited are unfortunately only available in German.	17-18

VERSION 2 - REVIEW

Reviewer 3

Name Kurth, Tobias

Affiliation Charité Universitätsmedizin Berlin, Institute of Public Health

Date 30-Sep-2026

COI TK reports having received research grants from the German Federal Joint Committee (G-BA) and personal compensation from the North-East German Society for Gynecological Oncology (NOGGO), AbbVie, the BMJ Group, and Frontiers Media S.A.

In this retrospective cohort analysis, Glaus and colleagues examined whether the appropriateness of comparators used in clinical trials supporting cancer drug indications is reflected in NCCN recommendation categories. The study included 110 indications comprising 176 patient populations. The topic is relevant, but I have several methodological concerns regarding the unit of analysis and interpretation of the statistical comparisons. I understand that this is a revised version of the manuscript and that I was not involved in the previous round of peer review.

1. The unit of analysis does not map cleanly onto the research question. Comparator status is defined at the population level. Still, the main analysis classifies indications by whether at least one population had an appropriate comparator, an inappropriate comparator, or a single-arm trial. Since an indication can belong to more than one comparator group, the same indication may contribute to several groups.

This makes the resulting comparisons difficult to interpret. A more direct approach would be to compare the NCCN category of each population according to the comparator used for that population, while accounting for clustering of populations within indications or drugs.

2. The bootstrap procedure needs clarification. The manuscript does not state what was resampled. Given that populations are clustered within indications and indications can contribute to several comparator groups, the resampling procedure should preserve this dependence structure. The authors should specify the resampling unit and how the group differences were recalculated within each bootstrap sample.

3. The interpretation of statistical significance is too strong. The difference between the appropriate- and inappropriate-comparator groups was 6 percentage points with a 95% CI from −11% to 25%. This interval is compatible with both little difference and a potentially meaningful difference. The finding should therefore not be interpreted as showing that comparator appropriateness was "not associated" with NCCN category 1 recommendations.

Similarly, the hematologic malignancy estimate of 18% had a 95% CI from −22% to 53%, indicating substantial uncertainty. I would recommend focusing on the estimated differences and confidence intervals rather than whether the interval includes the null value.

4. The Abstract and Conclusion need some qualification. The Results report that 56/110 indications (51%) had at least one population tested with an appropriate comparator, whereas the Conclusion states "less than half." This should be corrected.

More importantly, the conclusion that appropriate versus inappropriate comparator choice was "not associated" with NCCN category 1 recommendations is stronger than supported by the data. The confidence interval is too wide to exclude a potentially relevant difference.

5. The study population is restricted to indications for which the German Federal Joint Committee determined an appropriate comparator. The interpretation and generalizability of the findings should remain clearly limited to this selected cohort.

VERSION 2 - AUTHOR RESPONSE

Reviewer 3	Author response and changes made	Page number in revised paper
In this retrospective cohort analysis, Glaus and colleagues examined whether the appropriateness of comparators used in	Thank you very much for your comments and your insightful feedback.	

clinical trials supporting cancer drug indications is reflected in NCCN recommendation categories. The study included 110 indications comprising 176 patient populations. The topic is relevant, but I have several methodological concerns regarding the unit of analysis and interpretation of the statistical comparisons. I understand that this is a revised version of the manuscript and that I was not involved in the previous round of peer review.		
1. The unit of analysis does not map cleanly onto the research question. Comparator status is defined at the population level. Still, the main analysis classifies indications by whether at least one population had an appropriate comparator, an inappropriate comparator, or a single-arm trial. Since an indication can belong to more than one comparator group, the same indication may contribute to several groups. This makes the resulting comparisons difficult to interpret. A more direct approach would be to compare the NCCN category of each population according to the comparator used for that population, while accounting for clustering of populations within indications or drugs	We thank the reviewer for this comment. The analysis was conducted at the population level, but the wording ("drug indication assigned if at least one population…") may have been misleading. Each population was assigned to its comparator group (inappropriate comparator, appropriate comparator, single-arm trial, no comparator). We calculated the proportion of population classified in each NCCN category (1, 2A, 2B, 3) for each comparator group (inappropriate, appropriate, single-arm and no comparator) and then averaged these proportions across drug indication within each comparator group. Thus, each drug indication counts equally.<br><br>To clarify this point, we revised our *methods* section as follows:<br><br>"Each population was classified into one of four comparator groups: appropriate comparator, inappropriate comparator, single-arm trial, or no data available. Within each drug indication, we calculated the proportion of populations classified in NCCN category 1, 2A, 2B or 3 for each comparator group. Then, we averaged these proportions across drug indications within each comparator group. Thus, each drug indication contributed equally, irrespective of the number of populations. A drug indication could contribute to multiple comparator groups if its populations belong to different comparator groups. However, each population is counted only once within its respective comparator group."	8, 10, 11, 23, Figure 1, Figure S2

	We also revised our *results* section:<br><br>"For populations tested with an appropriate comparator, the average proportion classified in NCCN category 1 per drug indication was 46% (56 drug indications), compared with 40% for populations tested with an inappropriate comparator (35 drug indications), 3% for populations based on single-arm trials (30 drug indications) and 14% for populations with no data available (7 drug indications). For populations tested with an appropriate comparator, the average proportion classified in NCCN category 2A per drug indication was 52%, compared with 60% for populations tested with an inappropriate comparator. The proportion increased to 97% for populations based on single-arm trials and 86% for populations with no data available."<br>We also revised the *notes* of Figure 1:<br>"Percentages reflect the proportion of populations within a drug indication classified in each NCCN category for each comparator group and averaged across drug indications. Comparator groups refer to the comparator used for each population and are the following: appropriate comparator, inappropriate comparator, single-arm trials and no data available. A drug indication can contribute to more than one group."<br><br>Similarly, we modified the notes of Figure S2 in the appendix: "*Notes*: Percentages reflect the proportion of populations within a drug indication classified in each NCCN category for each comparator group and averaged across drug indications. Comparator groups refer to the comparator used for each population and are the following: appropriate comparator, inappropriate comparator, single-arm trials, and no data available. A drug indication can contribute to more than one group. For solid tumors, the difference in the average proportion of populations classified in NCCN category 1 between populations tested with an appropriate	

	and an inappropriate comparator was 1 percentage point (95% CI −22 to 18), and between populations tested with an appropriate comparator and populations based on a single-arm trial was 44 percentage points (95% CI 25 to 59). For hematologic malignancies, the corresponding differences were 18 percentage points (95% CI −22 to 53) and 36 percentage points (95% CI 10 to 67), respectively”	
2. The bootstrap procedure needs clarification. The manuscript does not state what was resampled. Given that populations are clustered within indications and indications can contribute to several comparator groups, the resampling procedure should preserve this dependence structure. The authors should specify the resampling unit and how the group differences were recalculated within each bootstrap sample.	We modified our methods to provide more details about the cluster bootstrap. We added the following information:<br><br>“95% confidence intervals were obtained by a nonparametric cluster bootstrap with drug indications as the resampling unit (1000 resamples; bias-corrected and accelerated intervals, R package boot). In each bootstrap sample, drug indications were drawn with replacement together with all their populations across comparator groups. Group averages and differences were recalculated. This allowed to preserve the correlation of NCCN categories among populations of the same drug indication as well as the dependence between comparator groups arising from drug indications contributing to more than one group."	9
3. The interpretation of statistical significance is too strong. The difference between the appropriate- and inappropriate-comparator groups was 6 percentage points with a 95% CI from −11% to 25%. This interval is compatible with both little difference and a potentially meaningful difference. The finding should therefore not be interpreted as showing that comparator appropriateness was “not associated” with NCCN category 1 recommendations.<br><br>Similarly, the hematologic malignancy estimate of 18% had a 95% CI from −22% to 53%, indicating substantial uncertainty. I would recommend focusing on the estimated differences and confidence intervals rather than whether the interval includes the null value.	We agree with the reviewer and revised our manuscript accordingly. We replaced ‘not associated’ and ‘does not depend’ with ‘there is no not statistically significant difference’ (or similar wording) in the abstract, results, discussion and conclusion.<br><br>The *abstract* was revised as follows: “The average proportion of populations classified in NCCN category 1 was 6 percentage points higher with an appropriate than with an inappropriate comparator (95% CI −11 to 25), and 43 percentage points higher than with single-arm trials (95% CI 28 to 58).” And “The proportion of NCCN category 1 recommendations did not differ significantly between populations tested with an appropriate and an inappropriate comparator but was	2, 10-14

	significantly lower for populations supported by single-arm trials."<br><br>The *result* section was revised as follows: "The average proportion of populations classified in NCCN category 1 was 6 percentage points higher in the appropriate comparator group than in the inappropriate comparator group (95% CI −11 to 25). This difference was not statistically significant. The proportion was 43 percentage points higher in the appropriate comparator group than in the single-arm trial group (95% CI 28 to 58). (…) For solid tumors, the difference in the average proportion of populations classified in NCCN category 1 between populations tested with an appropriate and an inappropriate comparator was 1 percentage point (95% CI −22 to 18), and between populations tested with an appropriate comparator and populations based on a single-arm trial 44 percentage points (95% CI 25 to 59). For hematologic malignancies, the corresponding differences were 18 percentage points (95% CI −22 to 53) and 36 percentage points (95% CI 10 to 67), respectively."<br><br>The *discussion* section was revised as follows: "We found no statistically significant difference in the average proportion of populations classified in NCCN category 1 between populations tested with an appropriate and an inappropriate comparator. However, we found a significant difference between populations tested with an appropriate comparator and populations based on single-arm trials." As well as "This could explain the absence of a statistically significant difference between populations using an appropriate and inappropriate comparators."<br><br>Finally, the *conclusion* section was revised as follows: "The proportion of NCCN category 1 recommendations did not differ significantly between populations tested with an appropriate	

	and an inappropriate comparator, whereas they were significantly lower for populations supported by single-arm trials."<br><br>Regarding the hematologic malignancy, we reported point estimates and CIs as differences in percentage points (6 pp, 95% CI −11 to 25, etc.) but did not add wording calling the result inconclusive or highly uncertain. Our statement is simply that the null hypothesis could not be rejected.	
4. The Abstract and Conclusion need some qualification. The Results report that 56/110 indications (51%) had at least one population tested with an appropriate comparator, whereas the Conclusion states "less than half." This should be corrected. More importantly, the conclusion that appropriate versus inappropriate comparator choice was "not associated" with NCCN category 1 recommendations is stronger than supported by the data. The confidence interval is too wide to exclude a potentially relevant difference.	We thank the reviewer for pointing this out. We changed "Less than half" to "approximately half" (51%) in the abstract, discussion and conclusion. As mentioned above, we also modified our conclusion. It now reads as such: "The proportion of NCCN category 1 recommendations did not differ significantly between populations tested with an appropriate and an inappropriate comparator, whereas they were significantly lower for populations supported by single-arm trials."	2, 10, 16
5. The study population is restricted to indications for which the German Federal Joint Committee determined an appropriate comparator. The interpretation and generalizability of the findings should remain clearly limited to this selected cohort.	We added the following sentence in our limitation section: "Thus, the findings of our study may not be generalizable to all NCCN recommendations."	16
